# Characteristics of anti-IL-17/23 biologics-induced interstitial pneumonia in patients with psoriasis

**DOI:** 10.1371/journal.pone.0245284

**Published:** 2021-01-07

**Authors:** Hanae Miyagawa, Hiromichi Hara, Jun Araya, Shunsuke Minagawa, Takanori Numata, Yoshinori Umezawa, Akihiko Asahina, Hidemi Nakagawa, Kazuyoshi Kuwano

**Affiliations:** 1 Division of Respiratory Diseases, Department of Internal Medicine, The Jikei University School of Medicine, Tokyo, Japan; 2 Department of Dermatology, The Jikei University School of Medicine, Tokyo, Japan; Nippon Medical School, JAPAN

## Abstract

**Objectives:**

Anti-IL-17/23 biologics are increasingly used to treat psoriasis. We aimed to elucidate characteristics of drug-induced interstitial pneumonia (DIIP) caused by anti-IL-17/23 biologics.

**Methods:**

We retrospectively analyzed the clinical data of psoriasis patients treated with anti-IL-17/23 biologics. Chest CT was performed to evaluate DIIP. Serum KL-6 levels were measured before treatment (baseline) and during treatment.

**Results:**

A total of 603 psoriasis patients were treated with anti-IL-17/23 biologics with mean follow-up of 21.1 months. Six patients developed DIIP at mean 14 months after initiation of the therapy. Older age, higher baseline KL-6 value and more frequent pre-existing IPs were associated with development of DIIP by univariate analysis. At the onset of DIIP, elevated serum KL-6 levels with concomitantly increased ground glass opacity (GGO) in Chest CT were demonstrated. DIIP was improved by only cessation of causative agents in five patients but steroid therapy was needed in one patient.

**Conclusions:**

DIIP is a plausible complication of anti-IL-17/23 biologics. Age, baseline KL-6 level and underlying IP could be the risk factors for DIIP development. Serum KL-6 levels and chest CT are useful for not only predicting but also detecting DIIP caused by anti-IL-17/23 biologics.

## Introduction

Psoriasis is a chronic inflammatory disease mainly affecting the skin [1]. Increasing evidence suggests that IL-17/23 axis plays important roles in the pathogenesis of psoriasis [1–5]. Biologics inhibiting IL-17/23 axis has been developed to treat psoriasis, and currently, three anti-IL-23 and three anti-IL-17 biological agents are available for psoriasis in Japan [6]. Ustekinumab (UST) is a monoclonal antibody targeting IL-12/23 p40, and Guselkumab (GUS) and Risankizumab(RIS) are monoclonal antibodies targeting IL-23 p19. Secukinumab (SEC) and ixekizumab (IXE) are monoclonal antibodies targeting IL-17A, and brodalumab (BRO) is a fully human monoclonal antibody targeting the IL-17 receptor A. All of them have demonstrated high efficacy for psoriasis in clinical settings [7].

Interstitial pneumonia (IP) is one of frequent adverse events induced by a variety of drugs [8]. KL-6 is a high molecular weight mucin-like glycoprotein produced by type 2 pneumocytes and respiratory bronchiolar epithelial cells [9]. KL-6 is overproduced in regenerative epithelial cells following epithelial cell damage during IP progression, hence, the serum KL-6 levels have been considered a representative biomarker for IPs including drug-induced IP (DIIP) [10, 11].

Incidence, severity and prognosis of DIIP depend on not only the type of drug but also host specific reaction [8, 12–14]. There are several case reports showing DIIP by anti-IL-17/23 biologics [15–19] (Two cases of DIIP reported by Kikuchi et al [17] are also included in this study). Recently, association of noninfectious pneumonia with UST use was reported in JAMA dermatology, and a new warning for DIIP by UST was issued [20]. Hence, careful attention should be paid for this adverse event during treatment with anti-IL-17/23 biologics. However, the clinical characteristics of DIIP caused by anti-IL-17/23 biologics including UST remain to be determined. The aim of this study is to clarify the clinical characteristics of DIIP caused by anti-IL-17/23 biologics, including incidence, severity, and prognosis based on evaluation of KL-6 levels and chest CT findings.

## Methods

### Study subjects

This study is a retrospective cohort study of confirmed cases of psoriasis treated with biologic agents targeting anti-IL-17/23 biologics at Jikei University Hospital from July 1, 2011 (07/01/2011) to August 31, 2019 (08/31/2019). Patients were eligible for the study if they met the following inclusion criteria 1) Age ≥ 20 years old, 2) Confirmed cases of psoriasis by specialists of the Japanese Dermatological Association, 3) Treated with one of anti-IL-17/23 biologics. The exclusion criteria were 1) Patients with progressive cancer or fatal disease. A total of 603 psoriasis patients (UST;256, GUS;53,SEC;183, IXE;39, BRO;72) were included in this study. This study was approved by the Ethics Committee of Jikei University (29–078 (8694)), and adhered to the tenets of the Declaration of Helsinki. Based on the ethical guidelines of Jikei University, informed consent was not necessary for this retrospective study, and we performed opt-out consent on the website of our hospital. All data were fully anonymized before statistical analysis. Patients’ medical records during July 1, 2011 (07/01/2011) to August 31, 2019 (08/31/2019) were accessed. Serum KL-6 levels were measured with a commercially available ELISA kit (Nanopia KL-6, 202138–005, Tokyo, Japan) before treatment (baseline) and during treatment. The intervals were determined by the attending doctors. Chest computed tomography (CT) was performed based on worsening of respiratory symptom and/or elevation of KL-6 levels. The chest CT findings of these patients were evaluated by two specialists of the Japanese Respiratory Society (H.H. and H.M.). The psoriasis area severity index (PASI) score and type of psoriasis were determined by specialists of the Japanese Dermatological Association (Y.U., H.N., and A.A.).

### Diagnosis of DIIP

Diagnostic criteria for drug-induced lung injury by Camus P et al. has been widely used in the clinical setting [21]. Accordingly, diagnosis of DIIP was made based on the diagnostic criteria as follows. (1)The patients were treated with anti-IL-17/23 biologics that are known to induce IP (2) IP have been reported to be induced by anti-IL-17/23 biologics. (3) Other causes could be ruled out. (4) IP was improved after anti-IL-17/23 biologics discontinuation.

### Diagnosis of underlying IP

To evaluate underlying IP in 603 patients, physical examination and/or chest radiograph and/or chest CT were performed before biologic treatment. Chest CT was performed in 453 patients. According to the patients’ requests or by concerns about radiation exposure, Chest CT was not performed in 150 patients. Among the 150 patients, chest radiograph was performed in 115 patients, which showed no interstitial shadow. Only physical examination was performed in 35 patients, presenting no sign of IP. Chest CT and radiograph were evaluated by two specialists of the Japanese Respiratory Society (H.H. and H.M.).

### Data collection

We retrospectively reviewed the medical records of the enrolled patients. Clinical characteristics including age, sex, BMI, disease duration, smoking history, underlying IP, type of psoriasis, prior biologic treatment, and treatment period were investigated.

### Statistical analysis

Clinical indices among patients treated with different anti-IL-17 biologics were compared using analysis of variance followed by Bonferroni’s post hoc comparison tests and chi-squared tests. Student’s t-tests and chi-squared tests were performed to compare DIIP group and non DIIP group. A two-sided p<0.05 was considered statistically significant. All statistical analyses were performed with StatView, version 5 (SAS Institute Inc., Cary, NC, USA).

## Results

### Clinical characteristics of all patients treated with anti-IL-17/23 biologics (Table 1)

**Table 1:** Clinical characteristics of all patients.

	**Total**		**UST**		**GUS**		**SEC**		**IXE**		**BRO**		
	**(n = 603)**		**(n = 256)**		**(n = 53)**		**(n = 183)**		**(n = 39)**		**(n = 72)**		**p value**
Age (years)	54.0±16.3		53.8±17.1		55.0±17.3		53.6±15.5		55.3±12.9		54.3±16.3		0.96
Male (%)	418 (69.3)		174 (68.0)		37 (69.8)		131 (71.6)		29 (74.4)		47 (65.3)		0.79
BMI	23.9±3.9		23.4±3.6		23.6±3.9		23.7±3.8§		26.7±4.6		24.3±4.6		<0.0001
Disease duration (years)	16.4±11.7		15.4±10.8		15.1±8.7		15.4±11.3		20.8±13.8		20.4±14.8		0.003
History of smoking (%)	198 (34.6)	unknown: n = 30	78 (31.6)	unknown: n = 9	11 (25)	unknown: n = 9	65 (36.7)	unknown: n = 6	17 (45.9)	unknown: n = 2	27 (39.7)	unknown: n = 4	0.2
Underlying IP(%)	18 (3.0)		7 (2.7)		1 (1.9)		6(3.3)		1 (2.6)		3(4.2)		0.95
Type of psoriasis													0.005
PsV	523	(86.7%)	237	(92.6%)	47	(88.7%)	148	(80.9%)	30	(76.9%)	63	(87.5%)	
PsA	61	(10.1%)	13	(5.1%)	3	(5.7%)	30	(16.4%)	8	(20.5%)	7	(9.7%)	
GPP	8	(1.3%)	1	(0.4%)	0	(0%)	4	(2.2%)	1	(2.6%)	2	(2.8%)	
PsE	0	(0%)	0	(0%)	0	(0%)	0	(0%)	0	(0%)	0	(0%)	
PsG	1	(0.2%)	1	(0.4%)	0	(0%)	0	(0%)	0	(0%)	0	(0%)	
PPP	7	(1.2%)	3	(1.2%)	3	(5.7%)	1	(0.5%)	0	(0%)	0	(0%)	
others	1	(0.5%)	1	(0.4%)	0	(0%)	0	(0%)	0	(0%)	0	(0%)	
Treatment period (months)	21.1±21.3		31.5±26.6		4.0±4.0		18.5±12.3		7.2±5.6		11.0±7.4		<0.0001
biologic treatment													
UST	256	(42.5%)											
GUS	53	(8.8%)											
SEC	183	(30.3%)											
IXE	39	(6.5%)											
BRO	72	(11.9%)											
Prior biologic treatment													
IFX	41	(6.8%)	18	(7.0%)	1	(1.9%)	17	(9.3%)	3	(7.7%)	2	(2.8%)	<0.0001
ADA	78	(12.9%)	25	(9.8%)	3	(5.7%)	41	(22.4%)	5	(12.8%)	4	(5.6%)	
UST	59	(9.8%)	0	(0%)	12	(22.6%)	41	(22.4%)	0	(0%)	6	(8.3%)	
GUS	1	(0.2%)	0	(0%)	0	(0%)	1	(0.5%)	0	(0%)	0	(0%)	
SEC	50	(8.3%)	4	(1.56%)	9	(17.0%)	0	(0%)	8	(20.5%)	29	(40.3%)	
IXE	3	(0.5%)	0	(0%)	1	(1.9%)	1	(0.5%)	0	(0%)	1	(1.39%)	
BRO	12	(2.0%)	0	(0%)	2	(3.8%)	3	(1.6%)	7	(17.9%)	0	(0%)	
Total	244	(40.5%)	47	(18.4%)	28	(52.8%)	104	(56.8%)	23	(59.0%)	42	(58.3%)	
KL-6 (U/mL)	283.8±247.9		271.8±206.1		290.7±227.6		284.1±303.5		302.7±172.5		292.8±216.1		0.95
DIIP	6	(1.0%)	3	(1.16%)	1	(1.89%)	2	(1.09%)	0	(0%)	0	(0%)	0.95

UST: Ustekinumab, GUS: Guselkumab, SEC: Secukinumab, IXE: Ixekizumab, BRO: Brodalumab, IFX: infliximab, ADA: Adalimumab.

IP: Interstitial pneumonia, DILI: Drug-induced lung injury.

KL-6: Krebs von den lungen-6, PsV: Psoriasis vulgaris, PsA: Psoriatic arthritis, GPP: Generalized pustular psoriasis, PsE: Erythrodermic psoriasis, PsG: Guttate psoriasis, PPP: Palmoplantar pustulosis.

A total of 603 psoriasis patients (UST;256, GUS;53,SEC;183, IXE;39, BRO;72) were treated with anti-IL-17/23 biologics with an average follow-up of 21.1 months. Seventy-four patients sequentially used two anti-IL-17/23 biologics, twenty three patients used three of them, and four patients used four of them. Disease duration of psoriasis was longer in BRO group compared to UST and SEC group. Treatment periods of UST group were longer than the other groups, and those of SEC group were longer than those of GUS and IXE. Prior biologic treatments were different among the groups. Other factors including age, ratio of male sex, history of smoking, underlying IP, baseline KL-6 and incidence of DIIP were not different among groups. (Difference of disease duration, treatment periods, and prior biologic treatments might be attributed to different timing of approval of each agent in Japan. BMI and types of psoriasis were different among the groups but no appropriate explanation existed).

### Backgrounds of patients with or without DIIP by anti-IL-17/23 biologics

Six patients (1.0%, UST;3, GUS;1, SEC;2) developed DIIP at average 14 months after initiation of the therapy. Backgrounds of patients without DIIP and with DIIP were shown in Table 2. Patients with DIIP were older than those without DIIP. Underlying IP was frequent in DIIP group (n = 4, 66.7%) compared to non-DIIP group (n = 14, 2.3%). Baseline KL-6 levels in DIIP group (1008.8±746.1) were higher than those without DIIP (274.7±222.8). Clinical characteristics of 18 patients with underlying IP were shown in Table 3. Connective tissue diseases were not complicated with the patients. Ground-glass and/or irregular linear (reticular) opacity in the bilateral lower lobes was characteristic of underlying IP as described in our previous work [22]. At DIIP presentation, systemic treatment for IP including glucocorticoids, immunosuppressive drugs, and drugs related to drug-induced IP were not used. Among 18 patients with underlying IP, the difference between baseline KL-6 levels in 4 patients who developed DIIP and those in 14 patients who did not develop DIIP was not significant (DIIP: 923±818, non DIIP:781±869, p = 0.77) Baseline KL-6 levels were not associated with development of DIIP in this population. Titers of ANA (anti-nuclear antibody) were less than or equal to 1:160, and anti-Jo-1 antibodies were negative. Other markers for connective tissue diseases and IP were not measured.

**Table 2:** Comparison of clinical characteristics of patients with or without DIIP.

	without DIIP		with DIIP		
	(n = 597)		(n = 6)		p value
Age (years)	53.8±16.3*		69.3±9.0*		0.02
Male (%)	414 (69.3)		4 (66.7)		0.89
BMI	23.9±4.0		23.3±3.0		0.73
Disease duration (years)	16.4±11.7		20.0±14.4		0.45
History of smoking (%)	194 (34.2)	unknown: n = 30	6 (66.6)		0.10
Underlying IP(%)	14 (2.3)*		4 (66.7)*		<0.0001
Type of psoriasis					0.99
PsV	520	(87.1%)	5	83.3(%)	
PsA	60	(10.1%)	1	(16.7%)	
GPP	8	(1.3%)	0	(0%)	
PsE	0	(0%)	0	(0%)	
PsG	1	(0.2%)	0	(0%)	
PPP	7	(1.2%)	0	(0%)	
others	1	(0.2%)	0	(0%)	
Treatment period(months)	21.2±21.3		14.0±9.0		0.41
biologic treatment					
UST	253	(42.3%)	3	(50%)	0.80
GUS	52	(8.7%)	1	(16.7%)	
SEC	181	(30.3%)	2	(33.3%)	
IXE	39	(6.5%)	0	(0%)	
BRO	72	(12.1%)	0	(0%)	
Prior biologic treatment					
IFX	40	(6.7%)	1	(16.7%)	1.00
ADA	77	(12.9%)	1	(16.7%)	
UST	58	(9.7%)	1	(16.7%)	
GUS	1	(0.17%)	0	(0%)	
SEC	49	(8.2%)	1	(16.7%)	
IXE	3	(0.5%)	0	(0%)	
BRO	12	(2.0%)	0	(0%)	
Total	240	(40.2%)	4	(66.7%)	
KL-6 (U/mL)	274.7±222.8		1008.8±746.1*		<0.0001

SEC: Secukinumab, BRO: Brodalumab, IXE: Ixekizumab.

IP: Interstitial pneumonia, BI: Brinkman index, PASI: Psoriasis area severity index, KL-6: Krebs von den lungen-6, PsV: Psoriasis vulgaris, PsA: Psoriatic arthritis, GPP: Generalized pustular psoriasis, PsE: Erythrodermic psoriasis, PsG: Guttate psoriasis, PPP: Palmoplantar pustulosis.

**Table 3:** Clinical characteristics of underlying IP.

No.	Age	Sex	complicated CTD	CT findings Distribution		GGO	Reticular opacity	Emphysema	Baseline KL-6 levels(U/ml)	biologictreatment	IP activity by the biologic	ANA(IF) (×)	anti-Jo-1 antibody	DIIP
1	77	M	non	Bilateral lower lobe	Peripheral	+	+	+	561	SEC		-	-	+
2	79	M	non	Bilateral lower lobe	Peripheral	+	+	+	351	SEC		40	-	
3	76	M	non	Bilateral lower lobe	Peripheral	-	+	+	734	SEC		-	-	
4	74	M	non	Bilateral lower lobe	Central	+	+	-	413	SEC	improved	160	-	
5	65	M	non	Bilateral lower lobe	Peripheral	+	+	-	174	SEC		40	N.A.	
6	72	M	non	Bilateral lower lobe	Peripheral	+	-	-	3656	SEC		160	-	
7	77	M	non	Bilateral lower lobe	Peripheral	-	+	+	771	BRO		40	-	
8	69	M	non	Bilateral lower lobe	Peripheral	+	-	-	447	BRO		-	-	
9	56	F	non	Bilateral lower lobe	Peripheral	+	-	-	1207	BRO		40	-	
10	81	M	non	Bilateral lower lobe	Peripheral	+	+	+	632	IXE		40	-	
11	72	M	non	Bilateral lower lobe	Peripheral	+	+	+	770	UST	improved	40	-	
12	64	F	non	Bilateral lower lobe	Central	+	-	-	2149	UST		N.A.	N.A.	+
13	77	F	non	Bilateral lower lobe	Peripheral	+	-	-	296	UST	improved	40	N.A.	
14	75	M	non	Bilateral lower lobe	Peripheral	+	+	+	467	UST		-	-	+
15	81	F	non	Bilateral lobe	Diffuse	+	+	-	356	UST	improved	-	N.A.	
16	73	M	non	Bilateral lower lobe	Peripheral	-	+	+	675	UST		-	-	
17	75	M	non	Bilateral lower lobe	Peripheral	-	+	+	452	UST		80	N.A.	
18	79	M	non	Bilateral lower lobe	Peripheral	+	+	+	517	GUS		-	-	+

CTD: Connective tissue disease, ANA: Anti-nuclear antibody, N.A.: Not available.

GGO: Ground Glass Opacity, RF: Reumatiod factor.

IF: Immunofluorescence.

### Clinical characteristics of DIIP

**Fig 1:**
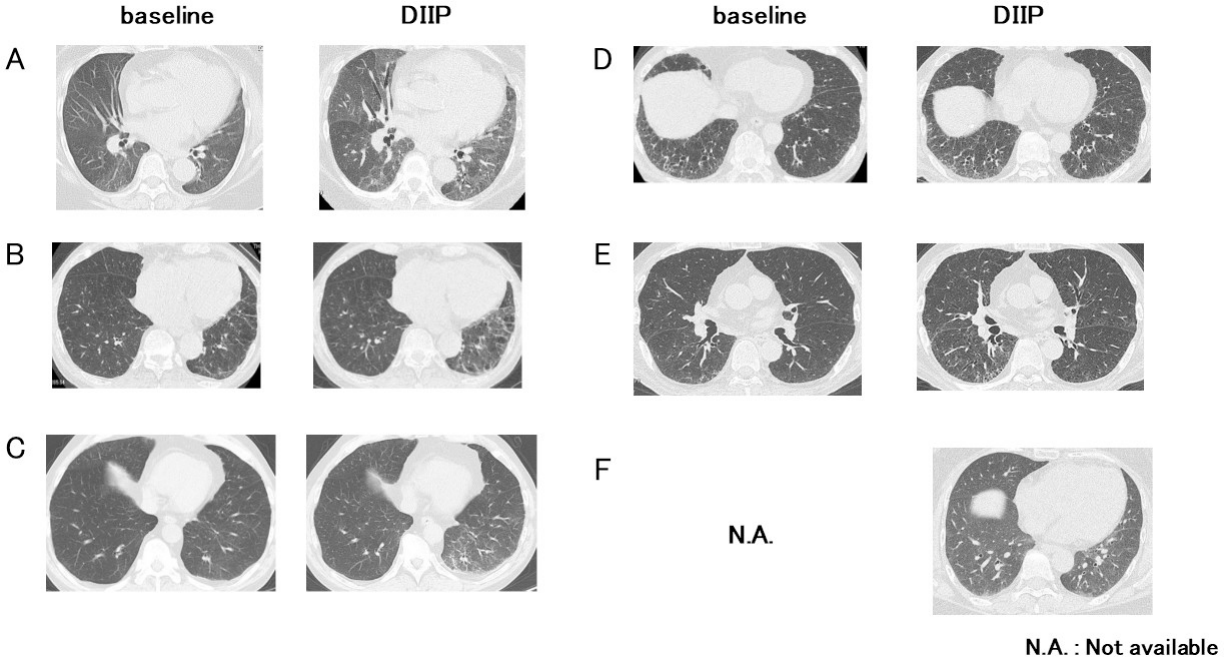
Chest CT in patients with DIIP. Panels of A-F demonstrated chest CTs of patient 1–6 shown in Tables 4 and 5. Each left panel showed chest CT before administration of causative biologic (baseline). Each right panel showed CT at the onset of DIIP. (Case 1 and 3 were identical with two cases in the Journal of Dermatology in 2016 [17]).

Clinical characteristics and course of DIIP were shown in Tables 4 and 5. (Among DIIPs patients, case 1 and 3 were already reported in J Dermatol. 2016 Jun;43(6):712–3.) Time to initial onset of DIIP ranged from 3 months to 26 months (average 14.0±9.0 months). While four of the six patients were symptomatic (cough or dyspnea), two patients were asymptomatic. In the asymptomatic patients, elevation of serum KL-6 levels from baseline was clue for further evaluation by CT scan, resulting in DIIP diagnosis. Chest CT findings at the onset of DIIP demonstrated patchy ground glass opacity (GGO) in all patients (Fig 1). Serum KL-6 levels were elevated at the onset of DIIP from baseline value, which were decreased after DIIP improvement. DIIPs were improved after cessation of the causative anti-IL-17/23 biologics without additional therapy in five patients but steroid therapy was needed in one patient. High baseline KL-6 levels and/or underlying IP were present in all patients with DIIP.

**Table 4:** Clinical characteristics of patients with DIIP (1).

No.	Age	Sex	BMI	Disease duration (year)	Smoking	type of Ps	Baseline KL-6 levels (U/ml)	underlying IP	prior biologic	Causative biologic of DIIP
1	64	F	27.4	1	-	PsV	2149	+	-	UST
2	75	M	19.5	30	+	PsV	467	+	-	UST
3	65	M	25.1	6	+	PsV	829	-	IFX	UST
4	79	M	21.3	22	+	PsV	332	+	SEC	GUS
5	77	M	21.3	20	+	PsV	561	+	UST	SEC
6	56	F	25.2	39	-	PsA	1715	-	ADA	SEC

**Table 5:** Clinical characteristics of patients with DIIP (2).

No.	Causative biologic	Time to initial onset of IP	symptom	hypoxia	CT findings	KL-6 Baseline	KL-6 onset of IP	KL-6 (after cessation of SEC)	Treatment for DIIP	Clinical course of IP
1	UST	3 M	cogh	+	GGO	2149	6809	2326	PSL	Improve
2	UST	17 M	cogh	-	GGO	467	1507	887	none	Improve
3	UST	26M	-	-	GGO	829	2092	991	none	Improve
4	GUS	12 M	-	-	GGO	332	755	448	none	Improve
5	SEC	21 M	dyspnea	-	GGO	561	1348	332	none	Improve
6	SEC	5 M	cough	-	GGO	1715	1800	614	none	Improve

GGO: Ground glass opacity.

## Discussion

In this study, we demonstrated that (1) incidence of DIIP by anti-IL-17/23 biologics was approximately 1.0% (6/603), (2) Age, baseline KL-6 level and underlying IP could be risk factors, (3) DIIP by anti-IL-17/23 biologics was mild, and developed at mean 14 months after initiation of the therapy (4) Serum KL-6 levels and chest CT were useful for not only predicting but also detecting DIIP.

Although many clinical studies have elucidated the efficacy and safety of anti-IL-17/23 biologics, no studies clearly demonstrated the incidence of DIIP, hence, our study is of great clinical importance. The actual incidence of DIIP was 1.0% of total patients in this study, which was similar to the incidence of DIIP by humanized biologics targeting TNF-alpha (0.5%) in Japanese postmarketing surveillance for Rheumatoid Arthritis [23].

Age, sex, smoking history, pre-existing lung diseases, and renal dysfunction are widely known risk factors for DIIP [8, 12, 13]. In this study, age, baseline KL-6 levels and pre-existing IP were suspected to be risk factors for DIIP for anti-IL-17/23 biologics by univariate analysis. (The number of DIIP cases was not enough for multivariate analysis.) Because KL-6 increases with IP, the effect of baseline KL-6 level on development of DIIP in this study might partly reflect the effect of underlying IP on development of DIIP. Indeed, baseline KL-6 levels did not show the significant effect on development of DIIP in patients with underlying IP. Larger studies are needed to determine the true risk factor of DIIP. Careful attentions for DIIP should be paid during anti-IL-17/23 biologics treatment in patients with these risk factors. In clinical practice, multiple different biologics were used sequentially in same patient and each biologics may have different effect on IP development. Increasing evidence suggests that anti-TNF-alpha biologics could increase KL-6 levels with or without clinical DIIP development or worsening of pre-existing IP [24]. It is plausible that increase KL-6 levels without IP progression may reflect subclinical alveolar inflammation caused by anti-TNF-alpha biologics. In case 3 and 6 in Tables 4 and 5 without pre-existing IP, KL-6 levels were elevated before anti-IL-17/23 biologics treatment, which might be associated with prior use of anti-TNF-alpha biologic. We speculate that elevated baseline KL-6 levels with subclinical alveolar inflammation by anti-TNF-alpha biologic pretreatment may enhance sensitivity to anti-IL-17/23 biologics-induced DIIP.

DIIP by anti-IL-17/23 biologics in this study was mild and with good prognosis. DIIPs were improved after cessation of the causative biologics without additional therapy in five patients but steroid therapy was needed in one patient in this study. In general, IP improvement by drug cessation without additional therapy can be an important clue for DIIP diagnosis (positive dechallenge). However, it is difficult to distinguish between DIIP development and worsening of pre-existing IP in the patient who needed additional steroid therapy. In our patient who was needed additional steroid therapy, pre-existing IP was stable for two year before administration of anti-IL-17/23 biologic. Furthermore, activity of psoriasis, which could be aligned with complicated IP activity [22], was markedly improved in response to anti-IL-17/23 biologics treatment, suggesting that DIIP is more likely than worsening of pre-existing IP.

Anti-IL-17/23 biologics could both improve and induce IP. Anti-IL-17/23 biologics improved IP in four (Characteristics of three patients were demonstrated in our previous report [22]) of 18 patients with underlying IP. IL-17/23 pathway is considered to be involved in the pathogenesis of the IP of the four patients. Activity of this type of IP was aligned with activity of psoriatic skin lesion, and both IP and skin lesions were improved within several months after first administration. DIIP by anti-IL-17/23 biologics, whose activity was not associated with activity of psoriatic skin lesion, developed later, even after two years as shown in this study. DIIP might be independent of IL-17/23 pathway and caused by other immunological mechanisms, which should be elucidated by further research.

There is a wide variation of clinical course and prognosis in DIIP, which is dependent on the type of causative agents. While immediate cessation of the causative agent and subsequent high dose of steroid therapy is needed in severe DIIP caused by EGFR tyrosine kinase inhibitors [25], continuation of the causative agent can be permissive in mild DIIP induced by mTOR inhibitors [26]. The results of this study and other previous reports of DIIP by anti-IL17/23 biologics [20] suggested that DIIP by anti-IIL-17/23 biologics can be mild with good prognosis. To clarify whether cessation is needed or not for mild DIIP by anti-IIL-17/23 biologics during treatment for refractory psoriasis, larger scale of clinical study should be conducted to determine the balance between treatment efficacy to psoriasis and severity of DIIP.

As shown in Table 5, it is likely that DIIP can occur at any time points during anti-IL-17/23 biologics treatments. DIIP could be occurred even at 26 months after first administration in patient 3, indicating that attentions for DIIP should be paid all the time during anti-IL-17/23 biologics treatment. Symptoms at the onset of DIIP were non-specific, hence, careful physiological, serological, and radiological evaluations are important for early diagnosis. At the onset of DIIP, serum KL-6 levels were elevated compared to the baseline and GGO in Chest CT emerged in all patients. Accordingly, monitoring of serum KL-6 levels before and during treatment and chest CT scan in case of KL-6 elevation could be useful for properly detecting DIIP.

## Conclusions

DIIP is a plausible side effect of anti-IL-17/23 biologics. Age, baseline KL-6 level and underlying IP might be risk factor for DIIP. Monitoring of serum KL-6 levels and chest CT scan might be useful for predicting and detecting DIIP.

## Abbreviations

ADA: adalimumab
BRO: brodalumab
CT: computed tomography
DIIP: drug-induced interstitial pneumonia
GUS: guselkumab
IFX: infliximab
IL-17/23: Interleukin-17/23
IP: interstitial pneumonia
IXE: ixekizumab
KL-6: Krebs von den Lungen-6
RIS: Risankizumab
SEC: secukinumab
TNF: tumor necrosis factor
UST: ustekinumab
